# Effectiveness of EMDR Therapy on Cognitive Performances in Patients with Post-Traumatic Stress Disorder and Complex Post-Traumatic Stress Disorder: A 6-Month Follow-Up Study

**DOI:** 10.1192/j.eurpsy.2025.508

**Published:** 2025-08-26

**Authors:** T. B. Jannini, L. Longo, R. Rossi, M. Gagliano, A. Pompeo, A. Di Mario, C. Niolu, A. Siracusano, G. Di Lorenzo

**Affiliations:** 1 Department of Experimental Medicine; 2 Department of Systems Medicine, Tor Vergata University of Rome, Rome, Italy

## Abstract

**Introduction:**

Complex post-traumatic stress disorder (cPTSD) is a clinical entity characterized not only by the typical symptoms of hyperarousal, avoidance, and flashbacks but also by disturbances in self-organization. Given the well-known association between trauma and cognitive deficits, it is common to observe a significant prevalence of such alterations among patients with cPTSD.

**Objectives:**

The aim of this study is therefore to assess cognitive functioning in response to treatment using Eye Movement Desensitization and Reprocessing (EMDR).

**Methods:**

Fifty-eight patients were recruited and divided into two groups (28 PTSD; 23 cPTSD), to whom scales for post-traumatic symptomatology (Impact of Event Scale – revised – IES-R; Clinician-Administered PTSD Scale – CAPS), along with cognitive tests (MATRICS Consensus Cognitive Battery - MCCB), were administered. The patients were evaluated at baseline (T0) and 6 months after the completion of the last EMDR session (T6).

**Results:**

EMDR was effective in the treatment of post-traumatic symptomatology (IES-R; CAPS - p<0.001). The PTSD group showed improvement in the domains of verbal learning (RAVLT), visual attention (TMT-A), and working memory (CBTT) (p<0.05). The cPTSD group reported improvement in the verbal learning domain (p<0.05).

**Conclusions:**

In addition to clinical symptomatology, EMDR has been shown to be effective in treating cognitive deficits in patients with PTSD and cPTSD. However, further studies are needed to confirm the results and identify the underlying mechanisms of this effect.

**Disclosure of Interest:**

None Declared

